# IL-15 armoring enhances the antitumor efficacy of claudin 18.2-targeting CAR-T cells in syngeneic mouse tumor models

**DOI:** 10.3389/fimmu.2023.1165404

**Published:** 2023-07-26

**Authors:** Hongtai Shi, Andi Li, Zhenyu Dai, Jiao Xue, Qi Zhao, Jiyuan Tian, Dandan Song, Hao Wang, Jianan Chen, Xiaokang Zhang, Kaisong Zhou, Huafeng Wei, Songbing Qin

**Affiliations:** ^1^ Department of Radiotherapy, The First Affiliated Hospital of Soochow University, Suzhou, China; ^2^ Department of Radiation Oncology, The Sixth Affiliated Hospital of Nantong University, Yancheng Third People’s Hospital, Yancheng, China; ^3^ Innovent Cells Pharmaceuticals, Inc., Suzhou, China; ^4^ Department of Radiology, The Sixth Affiliated Hospital of Nantong University, Yancheng Third People’s Hospital, Yancheng, China; ^5^ Innovent Biologics, Inc., Suzhou, China

**Keywords:** claudin 18.2, CAR T cells, IL-15, solid tumor, cellular therapy

## Abstract

Claudin 18.2 (CLDN18.2)-targeting chimeric antigen receptor (CAR)-modified T cells are one of the few cell therapies currently producing an impressive therapeutic effect in treating solid tumors; however, their long-term therapeutic efficacy is not satisfactory with a short duration of response. Transgenic expression of interleukin (IL)-15 has been reported to promote T-cell expansion, survival, and function and enhance the antitumor activity of engineered T cells *in vitro* and *in vivo*. Therefore, this study aimed to explore whether IL-15 modification would increase the antitumor activity of CLDN18.2-targeting CAR-modified T (CAR-T) cells in immunocompetent murine tumor models. CLDN18.2-specific CAR-T cells with (H9 CAR-IL15) or without transgenic IL-15 expression (H9 CAR) were generated by retroviral transduction of mouse splenic T cells. *In vitro*, compared with H9 CAR T cells, H9 CAR-IL15 T cells exhibited better expansion and viability in the absence of antigen stimulation, with a less differentiated and T-cell exhausted phenotype; although IL-15 modification did not affect the production of effector cytokines and cytotoxic activity in the short-term killing assay, it moderately improved the *in vitro* recursive killing activity of CAR-T cells against CLDN18.2-expressing tumor cells. *In vivo*, H9 CAR T cells showed no antitumor activity against CLDN18.2-expressing pancreatic tumors in immunocompetent mice without lymphodepleting pretreatment; however, H9 CAR-IL15 T cells produced significant tumor-suppressive effects. Furthermore, H9 CAR-IL15 T cells exhibited greater *in vivo* expansion and tumor infiltration when combined with lymphodepleting preconditioning, resulting in superior antitumor activity in two murine tumor models and a survival advantage in one tumor model. We further demonstrated that recurrent tumors following H9 CAR-IL15 T-cell therapy downregulated CLDN18.2 expression, suggesting immune escape through the selection of antigen-negative cells under persistent CAR-T-cell immune pressure. In conclusion, our findings provide preclinical evidence supporting the clinical evaluation of IL-15-expressing CLDN18.2 CAR-T cells in patients with CLDN18.2-positive tumors.

## Introduction

Gastric cancer (GC) and pancreatic adenocarcinoma (PAAD) are malignant tumors of the digestive system that seriously endanger human life and health. The latest global cancer statistics show that there will be approximately 1.089 or 0.495 million new cases of GC or PAAD worldwide in 2020, accounting for 5.6% or 2.6% of the global cancer incidence, and approximately 0.768 or 0.466 million deaths, accounting for 7.7% or 4.7% of the global cancer deaths (1). Despite treatment progress in recent years, drug resistance, recurrence, and metastasis are still inevitable (2, 3). The 5-year survival rate of patients with advanced GC is approximately 5%–20%, with a median survival time of 10 months (4). PAAD has a worse prognosis, with a 5-year survival rate of only 6%–9% (5). The poor prognosis of these two cancer types highlights the need for novel treatment approaches. One such option is adoptive cell transfer (ACT) of chimeric antigen receptor (CAR)-modified T (CAR-T) cells, a rapidly evolving immunotherapeutic approach with demonstrated efficacy in treating refractory/relapsed tumors, especially blood cancer (6).

Claudin 18.2 (CLDN18.2) is a stomach-specific isoform of claudin-18 and is thought to be an ideal therapeutic target for the treatment of advanced GC and PAAD due to its prevalent expression in both primary and metastatic GC and PAAD with limited expression in normal tissues (7), and recent success of the first-in-class CLDN18.2-targeting monoclonal antibody (mAb) zolbetuximab achieving the primary endpoint in a phase 3 trial as first-line treatment of CLDN18.2-positive HER2-negative locally advanced or metastatic GC or gastroesophageal junction cancer (GEJ) clinically validates the great value of CLDN18.2 targeting in treating CLDN18.2-positive cancer (8). Given the favorable expression profile, CLDN18.2 is also a promising target for the development of CAR-T products, as the most concerning “on-target off-tumor” effect of CAR-T therapy could be minimized (9). Accordingly, multiple CAR-T products targeting CLDN18.2 are being preclinically or clinically evaluated, among which the most advanced second-generation CAR-T product, CT041, developed by CARsgen Therapeutics, has been reported to induce a 48.6% overall response rate (ORR) in treating a total of 37 cases of CLDN18.2-positive cancer with a 57.1% ORR in patients with GC in an ongoing phase 1 clinical trial (10, 11). Despite this promising result, currently developing CLDN18.2-targeting CAR-T products still faces a long-standing unresolved issue when applying CAR-T treatment to solid tumors, i.e., the short-term duration of response in responding patients with a reported median progression-free survival (mPFS) of 4.2 months for CT041 therapy of GC (11). Therefore, developing next-generation CAR-T products targeting CLDN18.2 is mandatory and being actively pursued.

An armoring strategy with cytokines/chemokines and/or their receptors is thought to be a promising approach to enhancing the therapeutic efficacy of CAR-T cells in solid tumors (12). Regarding CLDN18.2-targeting CAR-T cells, Luo et al. demonstrated that concomitant expression of interleukin (IL)-7 and CCL21 could improve the antitumor efficacy of these CAR-T cells and even produce therapeutic effects without chemotherapy preconditioning in mouse tumor models, which is associated with increased survival and infiltration of armored CAR-T cells in tumors and possible elicitation of host antitumor immunity (13), and a clinical study of CAR-T products based upon this armored technology is ongoing. As a pleiotropic cytokine with an immune-potentiating effect on both T cells and natural killer (NK) cells, IL-15 armoring has also been reported to improve the antitumor activity of human CAR-T cells targeting multiple antigens across both blood and solid xenograft tumor models (14–18). Recently, Lanitis et al. elegantly demonstrated that mouse IL-15 modification confers tumor vasculature-targeted CAR-T cells with enhanced effector functions, engraftment, and tumor microenvironment (TME) reprogramming capability, leading to better tumor control in immunocompetent mice (19), further providing a robust rationale for developing IL-15-expressing CAR-T cells to improve the therapeutic activity of CAR-T cells in solid tumors. Herein, we developed CLDN18.2-targeting mouse CAR-T cells and explored their *in vitro* and *in vivo* antitumor activities and potential molecular mechanisms in immunocompetent mouse tumor models.

## Materials and methods

### Cell cultures

Panc02 mouse pancreatic tumor cells were provided by Dr. Long Qian (Cyrus Tang Haematology Center, Soochow University). B16F10 mouse melanoma cells, Hepa1-6 mouse hepatocellular carcinoma cells, and 293T cells were maintained by Innovent Cells Pharmaceuticals. Panc02 and B16F10 tumor cells have a C57BL/6 genetic background and were transduced by tricistronic lentiviruses encoding the claudin 18.1– or claudin 18.2–luciferase–green fluorescent protein (GFP) gene to stably express murine claudin 18.1 (Panc02-claudin 18.1 and B16F10-claudin 18.1 cell) or murine claudin 18.2 (Panc02-claudin 18.2 and B16F10-claudin 18.2 cell) and the GFP marker gene and luciferase gene, respectively, for cell sorting and luciferase-based cytotoxicity assay. Claudin 18.1- or claudin 18.2-expressing Panc02 and B16F10 tumor cells were further transduced with the luciferase–GFP-encoding lentivirus, and pooled stably transfected cells were used in the cytotoxicity assays. Panc02, Hepa1-6, and 293T cells were cultured in Dulbecco’s modified Eagle medium (DMEM) (Solarbio) supplemented with 10% fetal bovine serum (FBS) (ExCell Bio), 100 U/ml penicillin, and 100 µg/ml streptomycin sulfate (Gibco); B16F10 cells were maintained in Roswell Park Memorial Institute (RPMI) 1640 medium (Gibco) supplemented with 10% FBS, 100 U/ml penicillin, and 100 µg/ml streptomycin sulfate. Primary murine T cells were cultured in a T-cell medium (TCM) consisting of RPMI 1640 medium, 10% FBS, 100 U/ml penicillin; original typeset lacks of comma.

### CAR construction

The second-generation CAR consisted of the single-chain variable fragment (scFv) of the parental anti-claudin 18.2 69H9 clone (20), the murine CD8α hinge and transmembrane region, followed by the intracellular murine 4-1BB costimulatory signal domain (CSD) and CD3ζ stimulatory signal domain (SSD) without (H9 CAR) or with murine IL-15 (mIL-15) separated by the P2A self-cleaving peptide sequence (H9 CAR-IL15). The full-length codon-optimized genes encoding H9 CAR and H9 CAR-IL15 were synthesized by Genewiz and then cloned into the 5′ *Hin*dIII and 3′ *Nsi*I sites of the murine stem cell virus (MSCV) retroviral vector (Takara Bio). A non-targeting PG CAR-IL15 that specifically recognizes the IgG antibody with a mutant P329G Fc fragment was constructed by replacing the anti-claudin 18.2 scFv in H9 CAR-IL15 with anti-PG scFv (21).

### Retrovirus production

293T cells were transfected with a mixture of a CAR-expressing retroviral vector, a pCL-Eco retroviral packaging vector (plasmid #12371, Addgene), and polyethyleneimine (PEI; PolyScience). The mixture was prepared in Opti-MEM with a 5:1 ratio of PEI/plasmid and incubated for 30 min at room temperature; 48 h after transfection, retrovirus-containing supernatants were spun and filtered through a 0.45 µm syringe (Millipore). The collected virus supernatant was mixed with an equal volume of PEG 8000 (Sigma) overnight at 4°C. Then, the supernatant was removed by centrifugation, and the virus was resuspended by adding 1% T-cell culture medium at 4°C for 4–20 h, pooled, aliquoted, and stored at −80°C.

### T-cell isolation, activation, transduction, expansion, and phenotype assessment

Primary murine splenic CD3^+^ T cells were isolated from CD45.1 congenic C57BL/6 mice (Shanghai Model Organisms, China) according to the instructions of the EasySep Mouse T-Cell Isolation Kit (STEMCELL Technologies). Murine T cells were activated for 24 h with plated–coated anti-CD3 antibody (Ab; Thermo) plus soluble anti-CD28 Ab (eBioscience) and human IL-2 (hIL-2; Slpharm, Beijing) and then transduced with a CAR-encoding retrovirus in RetroNectin (Takara)-coated plates, followed by additional incubation for 48 h in the presence of hIL-2. Transduced T cells were cultured in TCM supplemented with hIL-2 from Day 2 with a medium change every 2–3 days. On Day 7 after transduction, CAR expression and the phenotype of the resultant CAR-T cells were assessed by flow cytometric analysis as described below, and the viability of the prepared CAR-T cells was analyzed by an acridine orange/propidium iodide (AO/PI) staining-based cell counter (Countstar Rigel S3).

### Cytokine release assay

A cytokine release assay was performed in triplicate by coculture-transduced T cells at an effector-to-target ratio of 1:1 for 24 h. The supernatant was harvested to test the secretion of cytokines. The secretion of interferon (IFN)-γ, tumor necrosis factor (TNF)-α, and IL-2 in the culture supernatants was measured by a Bio-Plex Pro Reagent Kit (Bio-Rad).

### Measurement of mIL-15

The mIL-15 in the supernatant or lysates was measured by an ELISA kit (Thermo Fisher). For intracellular mIL-15 protein quantification, transduced T cells were washed and placed in a cytokine-free medium. The next day, the T cells were harvested, lysed via one freeze/thaw cycle, and pelleted by centrifugation to harvest lysates. For the measurement of mIL-15 secretion by activated T cells, transduced T cells were stimulated with CD3/CD28 Ab for 24 h, and the supernatant was harvested for mIL-15 measurement by ELISA. For the measurement of mIL-15 in the supernatants from infected Hepa1-6 cells, Hepa1-6 cells were transduced with a CAR-encoding retrovirus in the presence of 8 μg/ml polybrene, and 24 h later, the medium was refreshed. Then, the resultant cells were assessed for CAR expression, and their supernatant was harvested for mIL-15 detection 72 h after infection.

### *In vitro* cytotoxicity assays

Triplicate wells of luciferase-expressing Panc02 or B16F10 tumor cells with stable expression of claudin 18.1 or claudin 18.2 were cocultured with mouse CAR-T cells at various E:T ratios for 24 h. The cytotoxicity of T cells was measured using the Bright-Glo™ luciferase assay system (Promega) according to the manufacturer’s instructions.

### *In vitro* serially dynamic killing assay

The dynamic killing activity of CAR-T cells against target cells was assessed by using a real-time impedance-based xCELLigence system (real-time cell analysis (RTCA) multiple-plate (MP) instrument, Agilent). First, the baseline measurement was settled by adding 50 µl of culture medium per well to E-plates (Roche, Switzerland). Then, 50 μl of culture medium containing target cells (15,000 Panc02-claudin 18.2 cells/well, 10,000 B16F10-claudin 18.2 cells/well) was added to E-plates per well, and electrical impedance was measured throughout the cultivation period at 15 min intervals until the target cells were in logarithmic growth; 20 h later, 50 μl of culture medium containing CAR-T cells with equal CAR positivity (adjustment by using untransduced T (UN-T) cells) was plated at a 1:3, 1:1, or 3:1 E/T ratio in E-plates in triplicate in each condition. The addition of culture medium or lysis solution served as the negative control (NC) and positive control (PC), respectively. Electrical impedance was measured for 72–96 h. In the second and the following round, CAR-T cells from the prior round were harvested, medium refreshed, and then transferred to a new E-plate with plated target cells without E/T ratio adjustment.

### Flow cytometry

Fluorochrome-conjugated isotype controls, fluorescein isothiocyanate (FITC) anti-mouse CD3ϵ (clone 17A2), BUV395 anti-mouse CD4 (clone RM4-5), BUV805 anti-mouse CD8α (clone 53-6.7), APC/Cyanine7 anti-mouse CD25 (clone 373), BUV661 anti-mouse CD69 (clone H1.2F3), Brilliant Violet 650™ anti-mouse/human CD44 (clone IM7), Brilliant Violet 510™ anti-mouse CD62L (clone MLE-14), PE anti-mouse CD215 (IL-15 receptor-α (IL-15Rα)) (clone 6B4C88), anti-mouse CD16/32 (clone 93), APC/Cyanine7 anti-mouse CD45.1 (clone A20), PE anti-mouse CD45.2 (clone 104), PE-CF594 anti-mouse CD279 (clone J43), PE anti-mouse TIGIT (clone A17200C), and Brilliant Violet 785™ anti-mouse TIM-3 (clone RMT3-23) were purchased from BD Biosciences or BioLegend (San Diego, CA). CAR expression was detected by staining with Biotin-SP (long spacer) AffiniPure F(ab')2 fragment goat anti-mouse IgG (Jackson ImmunoResearch) followed by secondary APC streptavidin (BioLegend). Flow cytometry data were acquired by BD FACSymphony A3 and analyzed by the FlowJo software (FlowJo, LLC).

### *In vivo* efficacy of CAR-T cells

All animal studies were conducted in the animal facility at Innovent Biologics with protocols approved by the Institutional Animal Care and Use Committees at Innovent Biologics. In the Panc02-claudin 18.2 pancreatic tumor model, CD45.2^+^ C57BL/6 recipient mice (6–8 weeks old, female, Vital River, Beijing, China) were inoculated subcutaneously with 4 × 10^6^ tumor cells in the right flank on Day 9 or 11 in the absence or presence of lymphodepletion pretreatment, and when the average tumor volume reached 80–100 mm^3^, they received one dose of intravenous (i.v.) infusion of 2.5 or 5 × 10^6^ CAR-T cells prepared from CD45.1^+^ congenic mice of the C57BL/6 genetic background with cyclophosphamide (CPA; Baxter Oncology GmbH) intraperitoneally (i.p.) administered at a dose of 150 mg/kg 1 day before CAR-T-cell infusion. In the B16F10-claudin 18.2 melanoma model, mice were inoculated subcutaneously with 4 × 10^5^ tumor cells in the flank of C57BL/6 mice on Day 11; when the average tumor volume reached 150 mm^3^, CPA was administered i.p. 1 day before i.v. infusion of 5 × 10^6^ CAR-T cells. In all experiments, tumor growth was measured by calipers twice a week, tumor volumes were calculated using the formula V = (L × W2)/2, and tumor growth inhibition (TGI) was defined as 1 − mean (TV_treat_)/mean (TV_control_). The mice were followed up for survival in one experiment.

### *In vivo* CAR-T detection

Peripheral blood and spleens were harvested from treated mice. A total of 30 µl of peripheral blood was then incubated in an antibody panel (CD3/CD45.1/CD45.2/CAR/IL-15Rα) at 4°C for 30 min, then red blood cells were lysed and fixed with red blood cell (RBC) lysis/fixation solution (BioLegend), and finally 10 µl of 123count beads (Thermo) was added for flow analysis and calculating the absolute number of CAR-T cells (expressed as CAR-T count per 100 μl blood). The spleens were gently crushed through a 40 μm cell strainer, followed by addition of ACK lysing buffer (Gibco) to make a cell suspension, then part of the resultant splenocytes were incubated in an antibody panel (CD3/CD45.1/CD45.2/CAR) and washed with FACS buffer twice, and finally 10 µl of 123count beads was added for flow analysis and calculating the absolute number of CAR-T cells.

### IHC analysis

Tumors were harvested from euthanized mice, fixed in 10% neutral buffered formalin, and processed for paraffin embedding. Paraffin sections were prepared using a microtome, and immunohistochemical (IHC) staining was performed. Primary antibodies used for IHC staining were anti-mouse CD3 antibody (clone E4T1B, Cell Signaling Technology), anti-mouse CD31 antibody (clone D8V9E, Cell Signaling Technology), and anti-mouse claudin 18 (Polyclone, Sigma) with secondary staining with a Discovery ultraMap Anti-Rb AP plus Discovery Red Kit (Roche) or a Discovery ultraMap anti-Rb HRP plus Discovery ChromoMap DAB Kit (Roche). Images were scanned with an Aperio Versa 8 (Leica Biosystems). For CD3 and claudin 18 staining, positive cells were quantified with the Halo image analysis software (Indica Labs); for CD31 staining, two to five representative views under 20× magnification were selected randomly in the live tumor area, the number of CD31^+^ blood vessels was counted, and the average blood vessel number was calculated.

### Statistical analysis

Data represent the mean ± SD from at least three biological replicates unless otherwise stated. Statistical analyses were performed using the GraphPad Prism 7 software. An unpaired two-tailed t-test was used for comparisons between two groups. For comparisons of three or more groups, the values were analyzed by one-way or two-way ANOVA with Bonferroni’s multiple-comparison correction. Mouse survival was analyzed by the Kaplan–Meier method and by the log-rank Mantel–Cox test. Statistical significance was defined as *, P < 0.05; **, P < 0.01; ***, P < 0.001; and ****, P < 0.0001.

## Results

### Generation and characterization of IL-15-expressing anti-CLDN18.2 CAR-T cells

**Figure 1:**
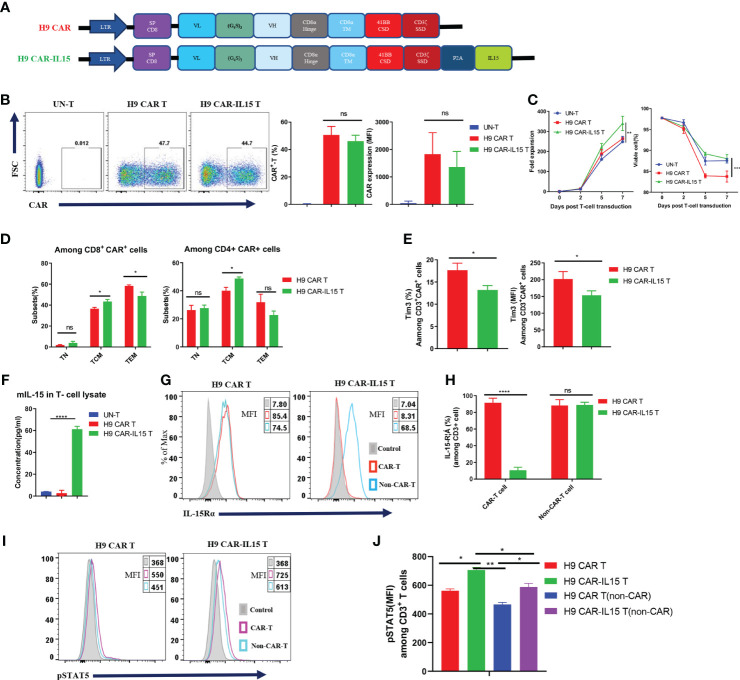
Generation and characterization of IL-15-expressing anti-CLDN18.2 CAR-T cells. **(A)** Schematic of retroviral vector constructs encoding H9 CAR or H9 CAR-IL15. **(B)** Representative flow cytometry plots of CAR expression on Day 7 after transduction (left), mean percentage and MFI of CAR expression pooled from four independent experiments (right). **(C)** Mean fold expansion of CAR-T cells or percentage of viability over time from four independent experiments. **(D)** Mean percentage of TN (CD44^−^CD62L^+^), TCM (CD44^+^CD62L^+^), and TEM (CD44^+^CD62L^-^) cells in the CD8 (left) or CD4 (right) subsets pooled from four independent experiments. **(E)** Mean percentage (left) or MFI (right) of TIM-3 expression in H9 CAR T and H9 CAR-IL15 T cells on Day 7 after transduction pooling from three independent experiments. **(F)** mIL-15 protein in the lysates of H9 CAR-IL15 T cells determined by ELISA. The results show the mean concentration (pg/ml) ± SD of triplicate wells from one representative of two independent experiments. **(G, H)** Representative flow cytometry plots **(G)** and mean percentage **(H)** of IL15-Rα expression on both CAR-T or non-CAR-T cells of H9 CAR or H9 CAR-IL15 T cells on Day 7 after transduction. Experiments were repeated three times with similar results. **(I, J)** Representative flow cytometry plots **(I)** and MFI **(H)** of IL15-Rα expression on both CAR-T or non-CAR-T cells of H9 CAR or H9 CAR-IL15 T cells on Day 2 after transduction. Experiments were repeated three times with similar results. Statistical analyses were performed using one-way ANOVA with Tukey’s *post-hoc* correction test **(B, F)**, two-way ANOVA **(C)**, and two-tailed unpaired Student’s t-test **(D, E, H, J)**. Statistical significance was defined as follows: ns, not significant, P > 0.05; *, P < 0.05; **, P < 0.01; ****, P < 0.0001.

We previously generated a panel of mouse anti-human CLDN18.2 (hCLDN18.2) mAbs by using conventional hybridoma technology. In this study, the parental sequence of the 69H9 clone was selected to construct CLDN18.2-targeting mouse CAR-T cells (20). The 69H9 mAb can specifically bind to hCLDN18.2 or mouse CLDN18.2 (mCLDN18.2) with high affinity but not human or mouse CLDN18.1 (hCLDN18.1 or mCLDN18.1), another isoform of CLDN18 expressed in normal lung epithelial cells, facilitating toxicological study in a mouse model. To construct second-generation mouse anti-CLDN18.2 CAR (H9 CAR), the scFv of the 69H9 clone was directly linked with the hinge and TM domain of the mouse CD8α molecule, CSD of the mouse 4-1BB molecule, and SSD of the mouse CD3ζ molecule in tandem; for the CAR with transgenic mIL-15 expression (H9 CAR-IL15), it further included a C-terminal mIL-15 sequence separated by a P2A self-cleavage sequence (**Figure 1A**). To generate mouse CAR-T cells, purified murine splenic CD3^+^ T cells were activated with αCD3/CD28 Ab and then transduced with a retrovirus encoding H9 CAR or H9 CAR-IL15 with UN-T cells as a control. As shown in **Figure 1B**, we consistently generated >40% CAR-positive mouse CAR-T cells with similar transduction efficiency for both CAR constructs (mean: 50.6% vs. 46.1%, n = 4 for H9 CAR vs. H9 CAR-IL15). Notably, H9 CAR-IL15 T cells exhibited greater *in vitro* fold expansion in culture (**Figure 1C**, left; mean: 339.3 vs. 263.9, n = 3, p < 0.01), probably due to their better maintained viability (**Figure 1C**, right; mean: 88.0 vs. 83.8, n = 3, p < 0.001) in culture. We also noticed that the viability of H9 CAR T cells was lower than that of UN-T cells, which may be caused by impurities present in the prepared retroviruses (**Figure 1C**, right). CAR transduction with or without IL-15 expression did not affect the CD4/8 T-cell subset composition, and CAR expression in these two subsets was similar (**Figures S1A, B**); however, H9 CAR-IL15 T cells had a higher percentage of CD44^+^CD62L^+^ central memory T cells (TCM) in both subsets (**Figures 1D** and **S1C**; mean: 48.7/43.4% vs. 40.0/36.5% in CD4/CD8 cells, n = 4 for H9 CAR vs. H9 CAR-IL15, p < 0.05). Furthermore, H9 CAR-IL15 T cells exhibited a less differentiated phenotype with lower levels of TIM-3 and TIGIT than H9 CAR T cells (**Figures 1E** and **S1D**). We detected little mIL-15 secretion in H9 CAR-IL15 culture supernatants, whereas significant mIL-15 was detected by ELISA after lysis of H9 CAR-IL15 T cells (**Figure 1F**), which was previously assumed to be due to mIL-15 sequestration by cell-surface IL-15Rα. This hypothesis was supported by the fact that we detected the notable expression of IL-15Rα on both CAR-T and non-CAR-T cells of H9 CAR T cells as well as non-CAR-T but not CAR-T cells of H9 CAR-IL15 T cells, as IL-15Rα detection on CAR-T cells of H9 CAR-IL15 T cells is likely to be blocked after binding of IL-15Rα to autocrine mIL-15 (**Figures 1G, H**); accordingly, we detected a significantly increased level of intracellular phosphorylated STAT5 (pSTAT5) in CAR-positive cells of H9 CAR-IL15 T cells compared with that of H9 CAR T cells (**Figures 1I, J**). In addition, we detected soluble mIL-15 in the supernatants of both transduced Hepa1-6 tumor cells and CD3/CD28 Ab-activated H9 CAR-IL15 T cells, whereas H9 CAR-IL15 T cells produced only moderate mIL-15 upon stimulation with CLDN18.2-expressing tumor cells (**Figures S1E–G**).

### *In vitro* antigen-specific recognition and effector functions of anti-CLDN18.2 CAR-T cells

**Figure 2:**
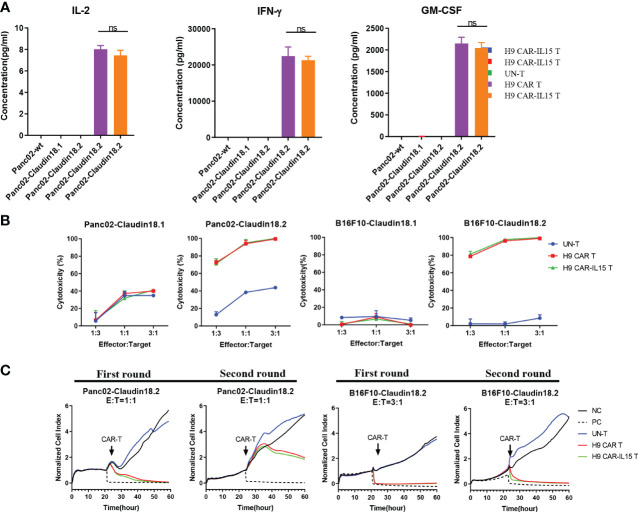
*In vitro* antigen-specific recognition and function of anti-CLDN18.2 CAR-T cells. **(A)** CAR-T cells were cocultured with different target cells at a 1:1 E:T ratio, and 24 h later, IFN-γ, IL-2, and GM-CSF production in the supernatants was determined by a Bio-Plex Pro Reagent Kit. The results show the mean concentration (pg/ml) ± SD of triplicate wells from one representative of three independent experiments. **(B)** CAR-T cells were cocultured with claudin 18.1- or claudin 18.2-expressing target cells at various E:T ratios for 24 h Cytotoxicity was measured using the Bright-Glo™ luciferase assay system. The results show the mean killing ± SD of triplicate wells from one of three independent experiments. **(C)** Dynamic killing of CAR-T cells against target cells was monitored by the xCELLigence RTCA MP instrument system for 72–96 h in each round. The experiment was repeated independently three times with similar results. Statistical analyses were performed using one-way ANOVA with Tukey’s *post-hoc* correction test **(A)**; statistical significance was defined as ns, not significant, P > 0.05. NC, negative control; PC, positive control.

We performed coculture assays of CAR-T cells with parental (Panc02-wt) or stably transfected Panc02 mouse pancreatic tumor cells expressing mCLDN18.2 (Panc02-claudin 18.2) or mCLDN18.1 (Panc02-claudin 18.1; **Figure S2A**). We did not observe that the expression of claudin 18.1 or claudin 18.2 conferred a proliferative advantage to Panc02 cells in *in vitro* assays (**Figure S2B**). As shown in **Figure 2A**, compared with UN-T cells, both H9 CAR and H9 CAR-IL15 T cells produced large amounts of IL-2, IFN-γ, and GM-CSF only in the presence of Panc02-claudin 18.2 cells, and the latter did not produce cytokines when stimulated by Panc02-wt and Panc02-claudin 18.1 cells, indicating antigen-specific recognition; little difference in cytokine production was observed between H9 CAR and H9 CAR-IL15 T cells. We next determined the *in vitro* killing activity of CAR-T cells against both Panc02 cells and B16F10 mouse melanoma cells expressing either CLDN18.2 (Panc02-claudin 18.2 cell and B16F10-claudin 18.2 cell) or CLDN18.1 (Panc02-claudin 18.1 cell and B16F10-claudin 18.1 cell; **Figure S2A**). Compared with UN-T cells, both H9 CAR and H9 CAR-IL15 T cells produced significant cytotoxic activity against both Panc02-claudin 18.2 and B16F10-claudin 18.2 cells (**Figure 2B**) but not Panc02-claudin 18.1 and B16F10-claudin 18.1 cells, indicating antigen-specific killing activity; similarly, we did not observe an appreciable effect of mIL-15 on the killing activity of CAR-T cells in this short-term killing assay. To test whether the benefits of mIL-15 expression need a longer time to manifest, we performed an *in vitro* recursive dynamic killing assay by using the xCELLigence system; in this setting, we found that H9 CAR-IL15 T cells exhibited slightly stronger and quicker killing activity against Panc02-claudin 18.2 and B16F10-claudin 18.2 cells than H9 CAR T cells, especially in the second round of stimulation (**Figure 2C**). However, this increase could not be maintained in further rounds, possibly due to the inability to accumulate sufficient concentrations of mIL-15 in our experimental conditions (**Figures S2C, D**).

### H9 CAR-IL15 T cells exhibit improved antitumor activity in immunocompetent murine tumor models

**Figure 3:**
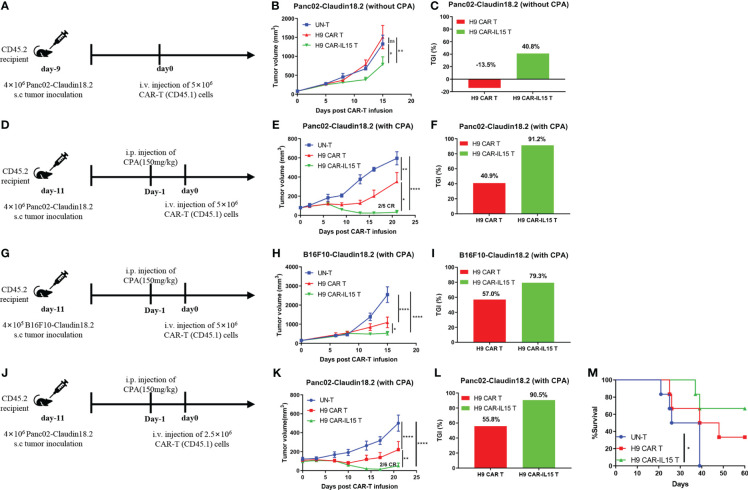
H9 CAR-IL15 T cells exhibit improved antitumor activity *in vivo*. **(A–C)** C57BL/6 mice were inoculated s.c. with Panc02-claudin 18.2 cells and treated with CAR-T cells in the absence of CPA pretreatment. **(A)** Experimental scheme of the *in vivo* antitumor experiment. **(B)** Tumor growth was assessed over time, and the results are expressed as the mean tumor volume (mm^3^ ± SD) with n = 5 mice per group. **(C)** The tumor growth inhibition in B is shown. **(D–F)** C57BL/6 mice were inoculated s.c. with Panc02-claudin 18.2 cells and treated with CAR-T cells in the presence of CPA pretreatment. **(D)** Experimental scheme of the *in vivo* antitumor experiment. **(E)** Tumor growth was assessed over time, and the results are expressed as the mean tumor volume (mm^3^ ± SD) with n = 5 mice per group. **(F)** The tumor growth inhibition of CAR-T cells in E is shown. **(G–I)** C57BL/6 mice were inoculated s.c. with B16F10-claudin 18.2 cells and treated with CAR-T cells in the presence of CPA pretreatment. **(G)** Experimental scheme of the *in vivo* antitumor experiment. **(H)** Tumor growth was assessed over time, and the results are expressed as the mean tumor volume (mm^3^ ± SEM) with n = 5 mice per group. **(I)** The tumor growth inhibition of CAR-T cells in H is shown. **(J–M)** C57BL/6 mice were inoculated s.c. with Panc02-claudin 18.2 cells and treated with CAR-T cells in the presence of CPA pretreatment. **(J)** Experimental scheme of the *in vivo* antitumor experiment. **(K)** Tumor growth was assessed over time, and the results are expressed as the mean tumor volume (mm^3^ ± SEM) with n = 6 mice per group. **(L)** The tumor growth inhibition in K is shown. **(M)** Survival of the mice was assessed. Statistical analyses were performed using two-way ANOVA **(B, E, H, K)**. Survival curves were analyzed by using a log-rank test **(M)**. Statistical significance was defined as follows: ns, not significant, P > 0.05; *, P < 0.05; **, P < 0.01; ****, P < 0.0001.

The *in vivo* efficacy of CAR-T cells with or without mIL-15 expression was evaluated in both Panc02-claudin 18.2 pancreatic tumor and B16F10-claudin 18.2 melanoma models. First, we tested the antitumor efficacy in the subcutaneous (s.c.) Panc02-claudin 18.2 pancreatic tumor in the absence of lymphodepleting pretreatment. In this model, we used *ex vivo* cultured Panc02-claudin 18.2 cells after one *in vivo* tumor passage, which contained approximately 90% CLDN18^+^ cells with the remaining CLDN18-negative cells, enabling us to explore any endogenous immunity induced by treatment (**Figure S3A**). Briefly, on Day 9 after tumor cell injection, with a tumor volume of ~80 mm^3^, CD45.2^+^ congenic C57BL/6 mice received one dose of intravenous infusion of 5 × 10^6^ CAR-T cells generated from CD45.1^+^ congenic mice (**Figure 3A**). As UN-T-cell treatment did not exhibit any antitumor effect compared with PBS treatment in this tumor model without or with CPA preconditioning (**Figures S3B, C**), we used UN-T-cell treatment as the control in the following experiments. As shown in **Figure 3B**, no tumor control was observed in mice treated with H9 CAR T cells compared with control treatment, while mice that received H9 CAR-IL15 T cells had significantly reduced tumor growth (mean tumor volume on Day 15 post CAR-T injection: 787.1 vs. 1,508.0 mm^3^, p < 0.01; **Figure 3C**, TGI: 40.8% vs. −13.5%, n = 5 for H9 CAR-IL15 vs. H9 CAR). Treatment with H9 CAR-IL15 T cells had improved antitumor activity; however, all treated mice eventually succumbed to tumor progression on Day 26 after treatment in this experiment.

To further enhance *in vivo* efficacy, tumor-bearing C57BL/6 mice received lymphodepleting preconditioning by i.p. administration of 150 mg/kg CPA 1 day before CAR-T infusion (**Figure 3D**) because previous studies have shown that CPA pretreatment enhances the antitumor efficacy of CAR-T cells by remodeling a favorable TME in multiple mouse tumor models, and CPA constitutes the cornerstone of the lymphodepleting regimen in the clinic (22, 23). As shown in **Figure 3E**, lymphodepleting preconditioning potentiated the antitumor efficacy of both CAR T-cell treatments; however, treatment with H9 CAR-IL15 T cells exhibited a better therapeutic effect than treatment with H9 CAR T cells (mean tumor volume on Day 21 post CAR-T injection: 30.8 vs. 351.8 mm^3^, p < 0.0001; **Figure 3F**, TGI: 91.2% vs. 40.9%, n = 5 for H9 CAR-IL15 vs. H9 CAR). Two mice (2/5) in this group achieved complete tumor regression (CR) on Day 21 after treatment; however, the tumor in one mouse began to recur on Day 27. When its tumor was analyzed on Day 47, we observed clear T-cell infiltration and loss of CLDN18.2 expression, whereas tumors treated with UN-T and H9 CAR T cells retained CLDN18.2 expression (**Figure S3D**). Furthermore, we obtained similar results in the more rapidly progressing B16F10-claudin 18.2 melanoma model by adopting the same treatment regimen (**Figures 3G, H**, mean tumor volume on Day 15 post CAR-T-cell injection: 525.5 vs. 1095.3 mm^3^, p < 0.0001; **Figure 3I**, TGI: 79.3% vs. 57.0%, n = 5 for H9 CAR-IL15 vs. H9 CAR).

To explore long-term therapeutic effects, we repeated the *in vivo* experiment in the Panc02-claudin 18.2 tumor model with the aim of testing different doses of CAR-T cells (**Figure 3J**). Treatment with a lower dose (2.5 × 10^6^) of H9 CAR-IL15 T cells still produced significantly better antitumor efficacy (**Figure 3K**, mean tumor volume on Day 21 post CAR-T injection: 51.3 vs. 221.0 mm^3^, p < 0.0001; **Figure 3L**, TGI: 90.5% vs. 55.8%, n = 5 for H9 CAR-IL15 vs. H9 CAR); at the end of the observation period (60 days after treatment), 66.7% (4/6) of the mice in this group remained alive, and the overall survival was significantly better than that of the control group (**Figure 3M**; p < 0.05).

The expression of IL-15 by H9 CAR-IL15 T cells is antigen independent, and one possible concern would be the induction of systemic toxicity by constitutive IL-15 secretion, as reported in a previous study describing severe toxicity induced by human IL-15-expressing CLL-1-targeting CAR-T cells in a human tumor xenograft model (18). As shown in **Figures S4A–D**, no significant body weight loss was observed in mice treated with H9 CAR-IL15 T cells compared with other groups in all but the B16F10-claudin 18.2 melanoma experiment where the decreased weight increment may be related with reduced tumor growth, which is further supported by the fact that no obvious pathologic damage could be detected in the vital organs from the mice receiving H9 CAR-IL15 T cells (**Figure S4E**). In addition, in an *in vivo* experiment including the treatment of non-targeting PG CAR-IL15 T cells, no significant body weight loss was seen in the mice of this group, although we indeed observed a trend toward slower tumor growth in those treated mice from Day 18 after treatment, which did not reach statistical significance (mean tumor volume on Day 21: 39.5 vs 454.1 or 301.7 mm^3^ for H9 CAR-IL15 vs UN-T or PG CAR-IL15, p < 0.0001; **Figure S4F**); this observation is possibly explained by the fact that IL-15 itself produced from non-activated CAR-T cells may induce the antitumor activity when it accumulates to a certain level, which is consistent with previous studies showing that IL-15 has modest anticancer activity as a single agent (24).

### Increased *in vivo* expansion and T-cell infiltration induced by H9 CAR-IL15 T cells

**Figure 4:**
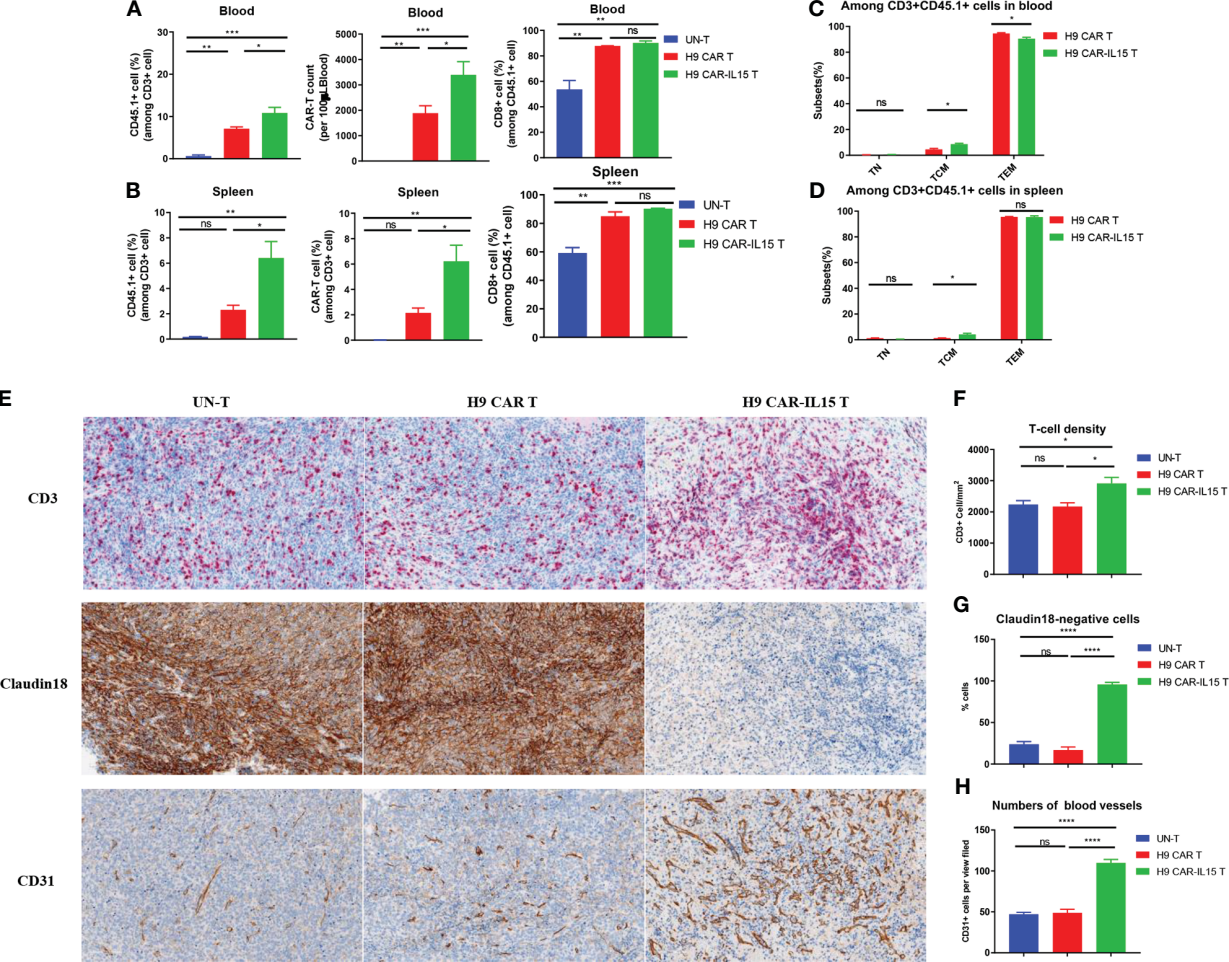
Increased *in vivo* expansion and T-cell infiltration induced by H9 CAR-IL15 T cells. **(A)** Abundance of transferred CD45.1^+^ T cells (left), the number of CAR-T cells, and percentage of CD8^+^ cells among CD45.1 T cells in blood from treated mice on Day 8 after CAR-T-cell treatment. The data represent the mean ± SD of three mice per group. **(B)** Abundance of transferred CD45.1^+^ T cells (left), the number of CAR-T cells (middle), and percentage of CD8^+^ cells (right) among CD45.1 T cells in spleens from treated mice on Day 8 after CAR-T-cell treatment. The data represent the mean ± SD of three mice per group. **(C)** Percentage of TN, TCM, and TEM cells among CD45.1^+^ T cells in the blood from treated mice on Day 8 after CAR-T-cell treatment. The data represent the mean ± SD of three mice per group. **(D)** Percentage of TN, TCM, and TEM cells among CD45.1^+^ T cells in spleens from treated mice on Day 8 after CAR-T-cell treatment. The data represent the mean ± SD of thee mice per group. **(E)** Representative IHC images at 200× magnification showing the expression of CD3 (upper), claudin 18 (middle), and CD31 (bottom) in tumors from UN-T (left), H9 CAR (middle), and H9 CAR-IL15-treated mice (right). **(F)** Mean density (per mm^2^) ± SD of CD3^+^ T cells in tumors from treated mice. **(G)** Mean percentage ± SD of claudin 18^+^ cells in tumors from treated mice. **(H)** Mean percentage (per view field) ± SD of CD31^+^ cells in tumors from treated mice. Statistical analyses were performed using one-way ANOVA with Tukey’s *post-hoc* correction test **(A, B, F, G, H)** and a two-tailed unpaired Student’s t-test **(C, D)**. Statistical significance was defined as follows: ns, not significant, P > 0.05; *, P < 0.05; **, P < 0.01; ***, P < 0.001; ****, P < 0.0001.

To elucidate the mechanism by which transgenic IL-15 expression improves the antitumor efficacy of CAR-T cells, we investigated the *in vivo* expansion and differentiation of adoptively transferred CAR-T cells. An *in vivo* experiment was performed as shown schematically in **Figure 3J**, in which some treated mice in each group were euthanized, and samples were collected for analysis on Day 8 after CAR-T-cell treatment. Compared with mice receiving H9 CAR T cells, mice treated with H9 CAR-IL15 T cells had a greater expansion of CAR-T cells *in vivo*, including their total T-cell percentages and absolute counts in peripheral blood and spleen (**Figures 4A, B**, **Figures S5A, B**), and the majority of CD45.1^+^ cells detected in mice receiving both CAR-T treatments were CAR-T cells, with the percentage of CD8^+^ subset predominating, consistent with target-induced preferential CAR-T expansion (**Figures S5A–D**); further phenotypic analysis revealed that expanding H9 CAR-IL15 T cells in both peripheral blood and spleen contained higher levels of CD44^+^CD62L^+^ TCM cells with correspondingly lower levels of CD44^+^CD62L^−^ TEM cells in peripheral blood (**Figures 4C, D**, **Figures S5E, F**). When we analyzed the subset changes and differentiation phenotypes of CD45.2^+^ host endogenous T cells in the peripheral blood and spleen, there were no significant differences between the two groups of treated mice (**Figures S5A–H**).

We next dissected tumor tissues by IHC staining for the expression of CD3, CD31, and CLDN18 biomarkers, indicative of intratumoral T-cell infiltration, angiogenesis, and antigen expression, respectively. As shown in **Figure 4E**, all tumors of treated mice exhibited significant T-cell infiltration on Day 8 after treatment; however, a significant increase in T-cell infiltration was observed in tumors treated with H9 CAR-IL15 T cells (**Figure 4F**). Accordingly, the expression of the target antigen CLDN18 was abolished only in tumors of this group, indicating that H9 CAR-IL15 T cells rapidly and efficiently engaged and killed antigen-positive tumor cells (**Figures 4E, G**). Unexpectedly, we observed enhanced angiogenesis in tumors treated with H9 CAR-IL15 T cells, as indicated by a marked increase in CD31^+^ angiogenic cells compared with other groups, suggesting that increased angiogenesis may benefit the function of CAR-T cells, at least in the Panc02 pancreatic tumor model (**Figures 4E, H**, **Figure S6**).

## Discussion

Although CAR-T therapy of hematological malignancies has achieved impressive efficacy in clinical settings, its efficacy remains limited in solid tumors due to multifactorial hurdles, such as insufficient T-cell tracking, tumor heterogeneity, inhibitory TME, and T-cell exhaustion; accordingly, versatile strategies have been proposed by recent investigations to combat those roadblocks, among which cytokine armoring is one of the promising avenues with multiple products in ongoing clinical trials. Here, considering the huge potential of CLDN18.2 as a clinically validated therapeutic target, we explored whether IL-15 armoring can improve the antitumor efficacy of second-generation CLDN18.2-targeting CAR-T cells and potential mechanisms in immunocompetent mice. We found that IL-15-expressing CAR-T cells display better *in vitro* expansion, possibly due to better retained viability and a “younger” phenotype with a greater fraction of TCM cells, which translates into better *in vitro* recursive killing activity and, importantly, more potent *in vivo* antitumor efficacy in two murine tumor models with an underlying mechanism involving the improved *in vivo* expansion and tumor infiltration of those cells. Our results provide a rationale for IL-15 armoring to potentiate the therapeutic effect of second-generation CLDN18.2-targeting CAR-T cells currently being tested in clinical trials.

*In vitro* characterization revealed the increased fold expansion and viability of H9 CAR-IL15 T cells compared with those of H9 CAR T cells at final harvest, with a less differentiated phenotype and expression of exhaustion-associated molecules, which is consistent with recent findings on the optimization of mouse CAR-T-cell preparations, although there is a difference between the studied CAR format (CD28 vs. 4-1BB domain) and cytokine used in culture (IL-7/IL-15 vs. IL-2) (19); thus, our study adds that IL-15 may exert similar phenotype-shaping effects on both CD28- and 4-1BB-contained CAR-T cells, although more studies are definitely needed to compare both at the transcriptional level. Regarding the effect of transgenic IL-15 on the phenotype of human T cells, previous studies have shown that IL-15 expression in CAR-T cells increases the proportion of less differentiated cells (naive T cells (TN), TCM, or stem memory T cells (TSCM)) and/or reduces the expression of exhaustion-associated molecules (PD-1 and LAG-3), although effects on CAR-T-cell expansion vary (14, 18); another study showed that IL-15-expressing human T cells maintain higher levels of CD27, CD28, and CD62L molecules in long-term culture without cytokine support compared with UN-T cells cultured with IL-2 or IL-15 (25). Thus, we speculate that the phenotypic effect would be more prominent if H9 CAR-IL15 T cells were grown in the absence of exogenous cytokines. Furthermore, most other studies have shown that human CAR-T cells expressing IL-15 maintain better survival after CAR engagement, with a higher proportion of TCM and/or TSCM memory cells and less expression of exhaustion-related molecules, especially after *in vitro* repeated stimulation (14–18, 25–27), which is most likely due to the emergence and accumulation of activation-induced IL-15 effects, as discussed below.

Consistent with previous studies, we did not detect the IL-15 in the culture supernatants of H9 CAR-IL15 T cells, which is coincident with an undetected expression of IL-15Rα on these cells, most likely due to IL-15 sequestration by IL-15Rα as previously described (19, 28); intriguingly, we found that IL-15Rα expression was detected on *in vitro* freshly prepared non-CAR- but not CAR-expressing H9 CAR-IL15 T cells, suggesting an autocrine rather than a paracrine effect of secreted IL-15, which is consistent with the increased pSTAT5 level detected in CAR-expressing H9 CAR-IL15 T cells. However, we detected only a low frequency of IL-15Rα^+^ circulating CAR-T cells or total infused T cells in treated mice, at the same frequency as host endogenous T cells (**Figure S7**), indicating upregulated IL-15Rα expression on freshly prepared CAR-T cells after *in vitro* activation returned to the resting level 1 week after CAR-T infusion. Future investigation of CAR-T cells with genetic reduction or elimination of IL-15R may clarify this issue more. When H9 CAR-IL15 T cells were stimulated by antigen-positive tumor cells, we detected only slight IL-15 production, whereas significant IL-15 was detected after CD3/CD28 bead stimulation, different from previous studies where significant IL-15 was detected by IL-15-expressing mouse or human CAR-T cells after cognate antigen stimulation (19). Unlike previous studies where the IL-15 gene was transduced as a separate vector or placed at the N-terminus of the CAR construct, we placed the IL-15 gene at the C-terminus of the CAR vector, which may impair the potency of IL-15 secretion, requiring more time (>24 h) or a stronger signal (repeated stimulation) to stimulate IL-15 production. Furthermore, given that IL-15 gene expression is highly regulated at multiple levels, it is also possible that differences in the IL-15 coding sequence caused our results to differ from others (25). This finding is also concordant with observations in studies of IL-15 transgenic human T cells that secrete little or low levels of IL-15 at resting conditions and preferentially produce high levels upon activation or repeated stimulation (15, 16, 28, 29), serving as an important safety feature since systemic administration of IL-15 has been associated with toxicities in human clinical studies (30, 31).

Consistent with previous studies of mouse and human engineered T cells with transgenic IL-15 expression (14–16, 18, 27, 29), our *in vitro* functional studies revealed that H9 CAR-IL15 T cells did not exhibit enhanced effector cytokine production and cytotoxic activity in short-term killing assays, whereas they showed an improved continuous killing capacity in serial killing assays, although the effect was weak and did not last more than three rounds, which may be due to suboptimal experimental conditions leading to insufficient accumulation and maintenance of the IL-15 effect. As mentioned above, this improved continuous killing capacity should be closely related to less exhaustion and better maintenance of memory cells in H9 CAR-IL15 T cells. The phenotypic and functional effect of IL-15 may be attributable to the upregulation of the anti-apoptotic molecule Bcl-2 and/or memory cell enhancer molecule TCF-1 by autocrine binding of IL-15, as previously described (17, 19). Future flow-based restimulation assay that simultaneously assesses CAR-T cell survival, proliferation, phenotype, and intracellular Bcl2 and TCF1 expression after each round of stimulation will allow us to answer this question.

Although the overall effect of IL-15 on CAR-T cells was generally insignificant in *in vitro* assays, its role *in vivo* is marked, resulting in the significantly improved antitumor activity of H9 CAR-IL15 T cells compared with that of unmodified CAR-T cells in both murine tumor models in the presence of lymphodepleting pretreatment. More impressively, even without lymphodepleting preconditioning, H9 CAR-IL15 T cells still produced a noticeable tumor-suppressing effect, while unmodified CAR-T cells had little effect, indicating that clinical application of IL-15-modified CAR-T cells may be feasible in the absence of a lymphodepleting regimen that generally incurs a remarkable toxic effect in cancer patients receiving CAR-T therapy (32). Mechanistic investigation showed that IL-15 expression induced greater *in vivo* expansion of CAR-T cells and tumor T-cell infiltration, which is likely related to the enhanced antitumor efficacy of H9 CAR-IL15 T cells, as previously described for IL-15-modified mouse and human engineered T cells (14–16, 18, 19, 27). Although we observed rapid CAR downregulation following CAR engagement *in vitro* (**Figure S8**), it is unlikely that this effect would compromise our accurate assessment of circulating CAR-T cells, as most of them may not be engaged, which is supported by our data showing that the majority of circulating CD45.1+ cells in mice receiving both CAR-T treatments were CAR^+^ cells (**Figure S5A**).

Interestingly, we found that Panc02-claudin 18.2 pancreatic tumors had a large number of intratumoral CD31^+^ angiogenic cells at early time points after H9 CAR-IL15 T-cell treatment, which is consistent with a recent study showing that treatment with IL-15-modified GD2-targeting CAR-T cells increased CD31^+^ angiogenic cells in orthotopic glioblastoma xenografts (33), suggesting that angiogenesis and possible normalization of vasculature may be related to antitumor activity, at least in some tumor models. The causal role of IL-15 in tumor angiogenesis treated with H9 CAR-IL15 T cells remains unclear; as previous studies have shown that IL-15 can promote angiogenesis or the production of angiogenic factors by cancer cells (34, 35), one possibility is that IL-15 secreted by activated H9 CAR-IL15 T cells promotes angiogenesis in treated tumors at early phase that may benefit the subsequent entry of CAR-T cells and host immune cells and ultimately contributes to the increased antitumor activity. We saw similar levels of vascularization in recurrent tumors as observed earlier in the H9 CAR-IL15 treatment group (**Figure S3D**). However, there was increased angiogenesis in tumors treated with UN-T and H9 CAR T cells, likely due to larger tumor sizes at later time points, obviating earlier observed differences in angiogenesis between groups; the reduction or even loss of IL-15 in recurrent tumors may not have maintained the differences in angiogenesis. In addition, we observed that almost all tumor cells lost expression of the target antigen on Day 8 after H9 CAR-IL15 T cell treatment, whereas most of tumor cells in the H9 CAR group, which exhibited comparable antitumor activity at this stage, maintained the expression of the target antigen; the underlying mechanism and significance of this observation remains unclear. We speculate that at an early phase, most of antigen-expressing tumor cells had been eliminated by H9 CAR-IL15 T cells, likely due to the hysteresis effect, making the difference in tumor sizes between two groups not significant. More studies are definitely needed to further clarify the molecular mechanism of IL-15 CAR-T-cell-induced increased angiogenesis and its potential involvement in antitumor activity, which will be investigated in more detail in our future studies.

Although transgenic IL-15 has been shown to increase the antitumor activity of CAR-T cells in multiple human CAR-T cells targeting various tumor antigens (14–16, 18, 33), a limitation of those studies is the use of xenograft models, which do not recapitulate the immunosuppressive tumor microenvironment created by tumors (36). In this regard, our study obliviates this weakness by evaluating the effect of IL-15 on CAR-T cells in immunocompetent mice, which also allows assessment of host endogenous immunity. Interestingly, in the Panc02-claudin 18.2 tumor model in which approximately 10% original tumor cells are negative for CLDN18.2 expression, we observed that some mice treated with H9 CAR-IL15 T cells eventually had relapsed tumor that lacked CLDN18.2 expression, indicating the occurrence of immune escape via selection of antigen-negative cells under continuous CAR-T-cell immune pressure, which is consistent with previous reports of IL-15-modified engineered T cells in human xenograft models (15, 27, 33). On the other hand, the emergence of antigen-negative tumor relapse indicates that treatment with H9 CAR-IL15 T cells in current regimens did not induce an effective host endogenous immune reaction to tumor cells independent of the targeted antigen in most treated mice, which is also concordant with our observation of minor changes in host immune cells in those mice. Hopefully, previous studies of mouse CAR-T cells have shown that lymphodepleting preconditioning or armoring strategies could effectively promote the induction of host endogenous antitumor immunity in some tumor models, resulting in long-term tumor protection (13, 22, 37, 38). Thus, it is possible that we can achieve the long-lasting antitumor efficacy by optimizing the treatment regimens or incorporation of further modifications.

Multiple IL-15-armored CAR-NK products have been tested in clinical trials with reported safety profiles in cancer patients (39). In our study, we also observed no apparent toxicity in mice treated with H9 CAR-IL15 T cells, which is consistent with previous studies of mouse and human CAR-T cells targeting CLDN18.2. However, a recent study of IL-15-expressing GPC3 CAR-T cells reported that infusion of those CAR-T cells into a patient with hepatocellular carcinoma (HCC) led to grade 4 cytokine release syndrome (CRS), which could be controlled by administration of rimiducid to trigger apoptosis of CAR-T cells (40). Thus, caution still should be taken before translation of IL-15-expressing CLDN18.2 CAR-T cells into the clinic. In this regard, incorporation of a safety switch in CAR design or full characterization of their safety features in preclinical studies should be beneficial.

In conclusion, our results suggested that the improved antitumor activity of IL-15-expressing CLDN18.2 CAR-T cells was mediated by their increased *in vivo* expansion and tumor infiltration promoted possibly by normalization of tumor vasculature. However, there is no doubt that more studies should be conducted to further elucidate the detailed mechanism as well as test the effects of transgenic IL-15 expression in human CLDN18.2 CAR-T cells. Our findings provide solid evidence for the clinical development of CLDN18.2 CAR-T cells as an immunotherapeutic strategy for CLDN18.2-expressing cancers.

## Data availability statement

The raw data supporting the conclusions of this article will be made available by the authors, without undue reservation.

## Ethics statement

The animal study was reviewed and approved by the Institutional Animal Care and Use Committees at Innovent Biologics.

## Author contributions

HS performed the experiments, analysed the data, wrote the manuscript and provided funding. JT and JC assisted with the mouse study and performed experiments. DS and HW performed IHC assays and analyses. AL and XZ provided materials and helped with the experimental design. ZD, JX, and QZ provided materials and funding. HFW conceptualized the study, designed experiments, analysed the data, and wrote the manuscript. SQ conceptualized the study and provided funding. All the authors reviewed/edited the manuscript. All authors listed have made substantial, direct, and intellectual contributions to the work and approved it for publication.

## Supplementary material

Supplementary Figure 1
**(A)** Representative flow cytometry plots of CD4 and CD8 expression in UN-T or transduced T cells on Day 7 after transduction (left) and mean percentage of CD4 and CD8 T cells from 4 independent experiments (right). **(B)** Representative flow cytometry plots of CAR expression on CD4 and CD8 T cells of UN-T or transduced T cells at Day 7 after transduction (left), mean percentage and MFI of CAR expression from 3 independent experiments (right). **(C)** Representative flow cytometry plots of CD44 and CD62L expression on UN-T or transduced T cells at Day 7 after transduction. **(D)** Representative flow cytometry plots of PD-1, TIGIT and TIM-3 expression on UN-T or transduced T cells at Day 7 after transduction (left) and mean percentage (middle) or MFI (right) of PD-1 and TIGIT expression in UN-T, H9 CAR T and H9 CAR-IL15 T cells at Day 7 after transduction. **(E)** Representative flow cytometry plots showing CAR expression on Hepa1-6 murine tumour cells transduced to express H9 CAR or H9 CAR-IL15 (left) and mIL-15 secretion in the supernatants from resultant cells with untransduced cells as controls. **(F)** mIL15 secretion in the supernatants of H9 CAR T and H9 CAR-IL15 T cells stimulated with CD3/CD28 Ab for 24 hours. **(G)** mIL15 secretion in the supernatants of Panc02-Claudin18.2 CAR T and H9 CAR-IL15 T cells with or without stimulation for 24 hours. The results show the mean concentration (pg/ml) ± SD of triplicate wells from one representative experiment. Statistical analyses were performed using one-way ANOVA with Tukey’s *post hoc* correction test **(A, B, E, F, G)** and a two-tailed unpaired Student’s t test **(D)**. Statistical significance was defined as follows: ns, not significant, P > 0.05; *, P < 0.05; **, P < 0.01; ***, P < 0.001; ****, P < 0.0001.

Supplementary Figure 2
**(A)** Representative flow cytometry plots showing the expression of Claudin 18.2 or GFP in stably transfected Panc02-Claudin 18.1, Panc02-Claudin 18.2, B16F10-Claudin 18.1, and B16F10-Claudin 18.2 cell lines. **(B)** Mean fold expansion of Panc02-wt and Panc02-Claudin18.2 cells over time from 3 independent experiments. **(C)** Serially dynamic killing activity of CAR-T cells against Panc02-Claudin 18.2 target cells was monitored by the Xcelligence RTCA MP instrument system for 72-96 hours in each round for a total of 2 rounds. **(D)** Serially dynamic killing activity of CAR-T cells against B16F10-Claudin 18.2 target cells was monitored by the Xcelligence RTCA MP instrument system for 72-96 hours in each round for a total of 3 rounds. The experiment was repeated independently 3 times with similar results. NC, negative control; PC, positive control.

Supplementary Figure 3
**(A)** Representative flow cytometry plots showing the expression of Claudin 18.2 and GFP in ex vivo cultured Panc02-Claudin 18.2 cells after one *in vivo* tumour passage. **(B, C)** C57BL/6 mice were inoculated s.c Panc02-Claudin18.2 cells and treated with 5 x 10^6^ UN-T cells or PBS (same volume) in the absence **(B)** or presence **(C)** of CPA pretreatment. Tumour growth was assessed over time, and the results are expressed as the mean tumour volume (mm3 ± SD) with n = 5 mice per group. **(D)** Representative IHC images and statistical results (bottom panel) for the expression of CD3 (upper panel), CD31 (lower panel) and Claudin 18 (middle panel) in tumours from UN-T (left), H9 CAR (middle) and H9 CAR-IL15 (right)-treated mice on Days 38 or 47 after treatment. Statistical analyses were performed using one-way ANOVA with Tukey’s *post hoc* correction test **(D)** and two-way ANOVA **(B, C)**. Statistical significance was defined as follows: ns, not significant, P > 0.05; *, P < 0.05; ****, P < 0.0001.

Supplementary Figure 4
**(A-D)** Body weight changes in treated mice over time were assessed. **(E)** Histopathological analysis of vital organ tissues by haematoxylin and eosin (H&E) staining of Panc02-Claudin18.2 xenografts pretreated with CPA. Mice were treated with CAR-T cells and sacrificed on Day 23 following T-cell infusion, and their vital organs were harvested, formalin-fixed, paraffin-embedded, and stained with H&E. Representative photomicrographs are shown. The images were taken at 200× magnification. **(F)** C57BL/6 mice were inoculated s.c. Panc02-Claudin18.2 cells and treated with CAR-T cells (2.5*10^6^ cells) in the presence of CPA pretreatment. Tumour growth was assessed over time, and the results are expressed as the mean tumour volume (mm^3^ ± SD) with n = 5 mice per group (UN-T group, PG CAR-IL15 T group, H9 CAR-IL15 T group). Body weight changes in treated mice over time were also assessed. Statistical analyses were performed using one-way ANOVA with Tukey’s *post hoc* correction test **(F)** and two-way ANOVA **(C)**. Statistical significance was defined as follows: ns, not significant, P > 0.05; *, P < 0.05; ***, P < 0.001.

Supplementary Figure 5
**(A, B)** Representative flow cytometric plots showing the percentage of CD45.1+ adoptively transferred cells and CD45.2+ host endogenous and CAR-T cells in the blood **(A)** and spleen **(B)** from treated mice at Day 8 after CAR-T-cell treatment. **(C, D)** Representative flow cytometric plots showing the percentage of CD8^+^ CAR-T (gating CD45.1^+^ cells) or host endogenous (gating CD45.2^+^ cells) T cells in the blood **(C)** and spleen **(D)** from treated mice at Day 8 after CAR-T treatment. **(E, F)** Representative flow cytometric plots showing the expression of CD44 and CD62L on CAR-T (gating CD45.1^+^ cells) or host endogenous (gating CD45.2^+^ cells) T cells in the blood **(E)** and spleen **(F)** from treated mice at Day 8 after CAR-T treatment. **(G)** Bar graphs showing the percentage of host endogenous CD45.2^+^ T cells and CD8^+^ T cells in the blood (left) and spleen (right) from treated mice at Day 8 after CAR-T-cell treatment. **(H)** Bar graphs showing the percentage of host endogenous TN (CD44^-^CD62L^+^), TCM (CD44^+^CD62L^+^), TEM (CD44^+^CD62L^-^) and TE (CD44^-^CD62L^-^) subsets in the blood (left) and spleen (right) from treated mice at Day 8 after CAR-T-cell treatment. The data represent the mean ± SD of 3 mice per group. Statistical analyses were performed using one-way ANOVA with Tukey’s *post hoc* correction test **(G, H)**. The significance of findings was defined as follows: ns, not significant; ns, P>0.05; *, P<0.05; **, P<0.01; ***, P<0.001.

Supplementary Figure 6 Full IHC images of tumours with two tumours each group for analysis of CD3, Claudin18.2 and CD31 expression.

Supplementary Figure 7 The bar graphs showing the percentage and/or MFI of IL15Rα expressed on circulating CAR-T cells (CD3^+^CD45.1^+^CAR^+^ cells), total infused T cells (CD3^+^CD45.1^+^ cells) and host endogenous T cells (CD3^+^CD45.2^+^ cells) from treated mice at day 7 after treatment. The data represent mean ± SD of 3 mice per group. Statistical analyses were performed using a one-way ANOVA with Tukey *post hoc* correction test.

Supplementary Figure 8 Effector cells (H9 CAR-IL15 T or PG CAR-IL15 T) and target cells (Panc02-Claudin18.2 or Panc02-Claudin18.1) were cocultured at 1:1 E/T ratio, 4 hours and 24 hours later, the expression of CAR on effector cells was detected by flow cytometry. The bar graphs showing the mean of relative CAR expression after CAR engagement (cocultured with Panc02-Claudin18.2) at 4 hours and 24 hours normalized to that after no CAR engagement (cocultured with Panc02-Claudin18.1). Results show mean ± SD of triplicate wells.
