# Supplementary figures and images for: IL-15 armoring enhances the antitumor efficacy of claudin 18.2-targeting CAR-T cells in syngeneic mouse tumor models
Source: Front Immunol. 2023 Jul 26;14:1165404. doi: 10.3389/fimmu.2023.1165404 (PMC10410263; doi:10.3389/fimmu.2023.1165404)

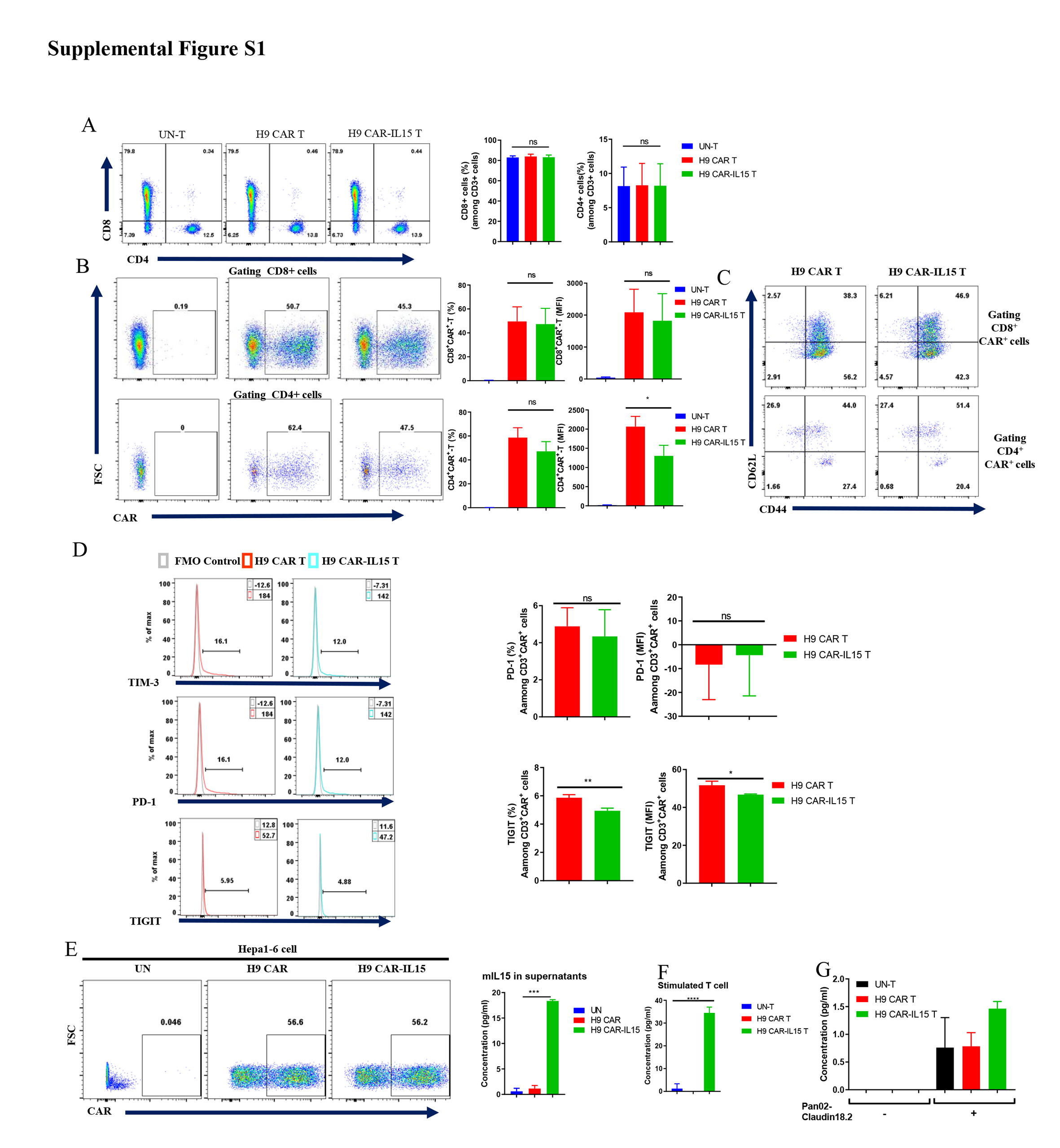

Supplement: Supplementary Figure 1 — (A) Representative flow cytometry plots of CD4 and CD8 expression in UN-T or transduced T cells on Day 7 after transduction (left) and mean percentage of CD4 and CD8 T cells from 4 independent experiments (right). (B) Representative flow cytometry plots of CAR expression on CD4 and CD8 T cells of UN-T or transduced T cells at Day 7 after transduction (left), mean percentage and MFI of CAR expression from 3 independent experiments (right). (C) Representative flow cytometry plots of CD44 and CD62L expression on UN-T or transduced T cells at Day 7 after transduction. (D) Representative flow cytometry plots of PD-1, TIGIT and TIM-3 expression on UN-T or transduced T cells at Day 7 after transduction (left) and mean percentage (middle) or MFI (right) of PD-1 and TIGIT expression in UN-T, H9 CAR T and H9 CAR-IL15 T cells at Day 7 after transduction. (E) Representative flow cytometry plots showing CAR expression on Hepa1-6 murine tumour cells transduced to express H9 CAR or H9 CAR-IL15 (left) and mIL-15 secretion in the supernatants from resultant cells with untransduced cells as controls. (F) mIL15 secretion in the supernatants of H9 CAR T and H9 CAR-IL15 T cells stimulated with CD3/CD28 Ab for 24 hours. (G) mIL15 secretion in the supernatants of Panc02-Claudin18.2 CAR T and H9 CAR-IL15 T cells with or without stimulation for 24 hours. The results show the mean concentration (pg/ml) ± SD of triplicate wells from one representative experiment. Statistical analyses were performed using one-way ANOVA with Tukey’s post hoc correction test (A, B, E, F, G) and a two-tailed unpaired Student’s t test (D). Statistical significance was defined as follows: ns, not significant, P > 0.05; *, P < 0.05; **, P < 0.01; ***, P < 0.001; ****, P < 0.0001. [file Image_1.tif]

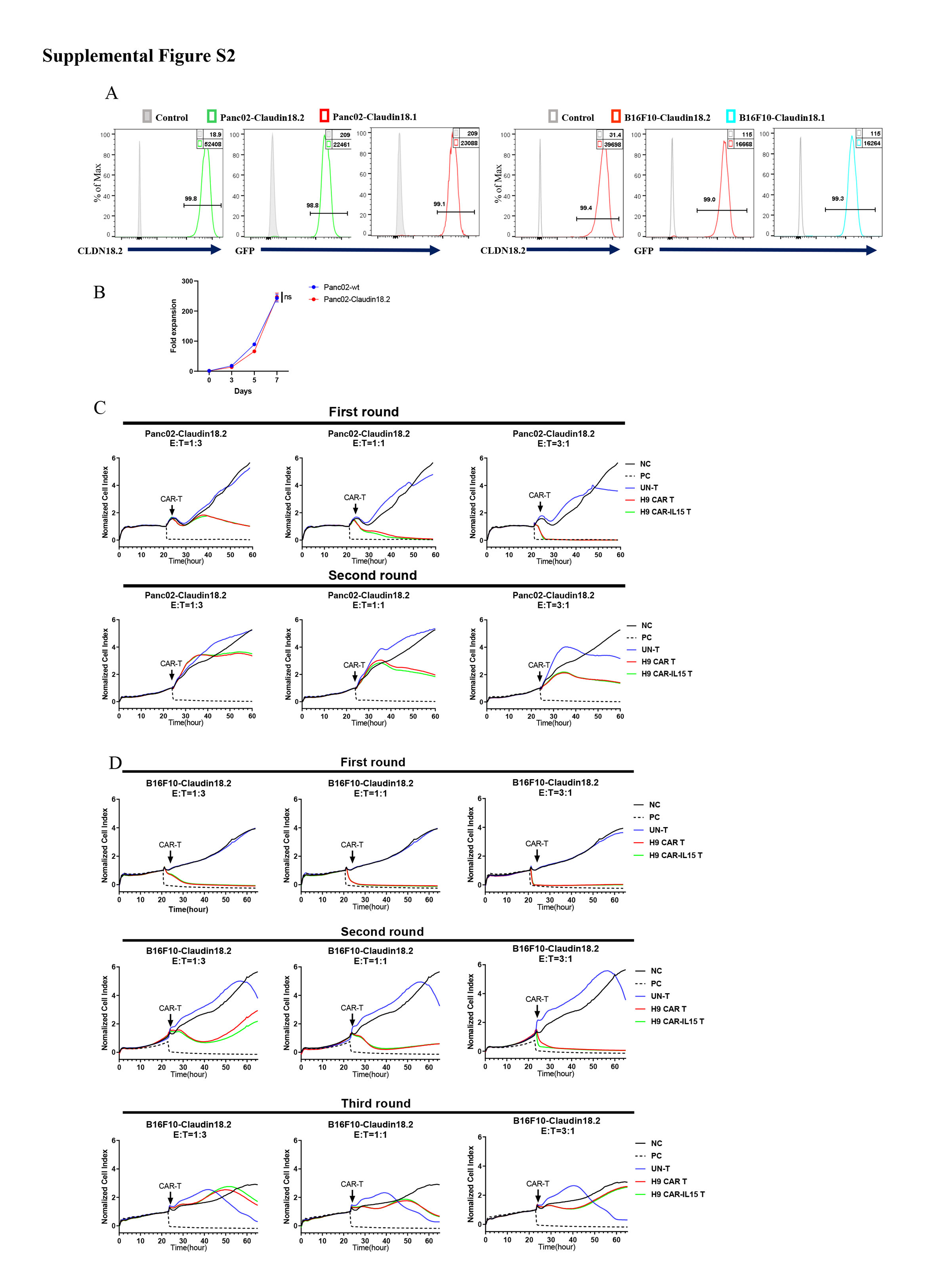

Supplement: Supplementary Figure 2 — (A) Representative flow cytometry plots showing the expression of Claudin 18.2 or GFP in stably transfected Panc02-Claudin 18.1, Panc02-Claudin 18.2, B16F10-Claudin 18.1, and B16F10-Claudin 18.2 cell lines. (B) Mean fold expansion of Panc02-wt and Panc02-Claudin18.2 cells over time from 3 independent experiments. (C) Serially dynamic killing activity of CAR-T cells against Panc02-Claudin 18.2 target cells was monitored by the Xcelligence RTCA MP instrument system for 72-96 hours in each round for a total of 2 rounds. (D) Serially dynamic killing activity of CAR-T cells against B16F10-Claudin 18.2 target cells was monitored by the Xcelligence RTCA MP instrument system for 72-96 hours in each round for a total of 3 rounds. The experiment was repeated independently 3 times with similar results. NC, negative control; PC, positive control. [file Image_2.tif]

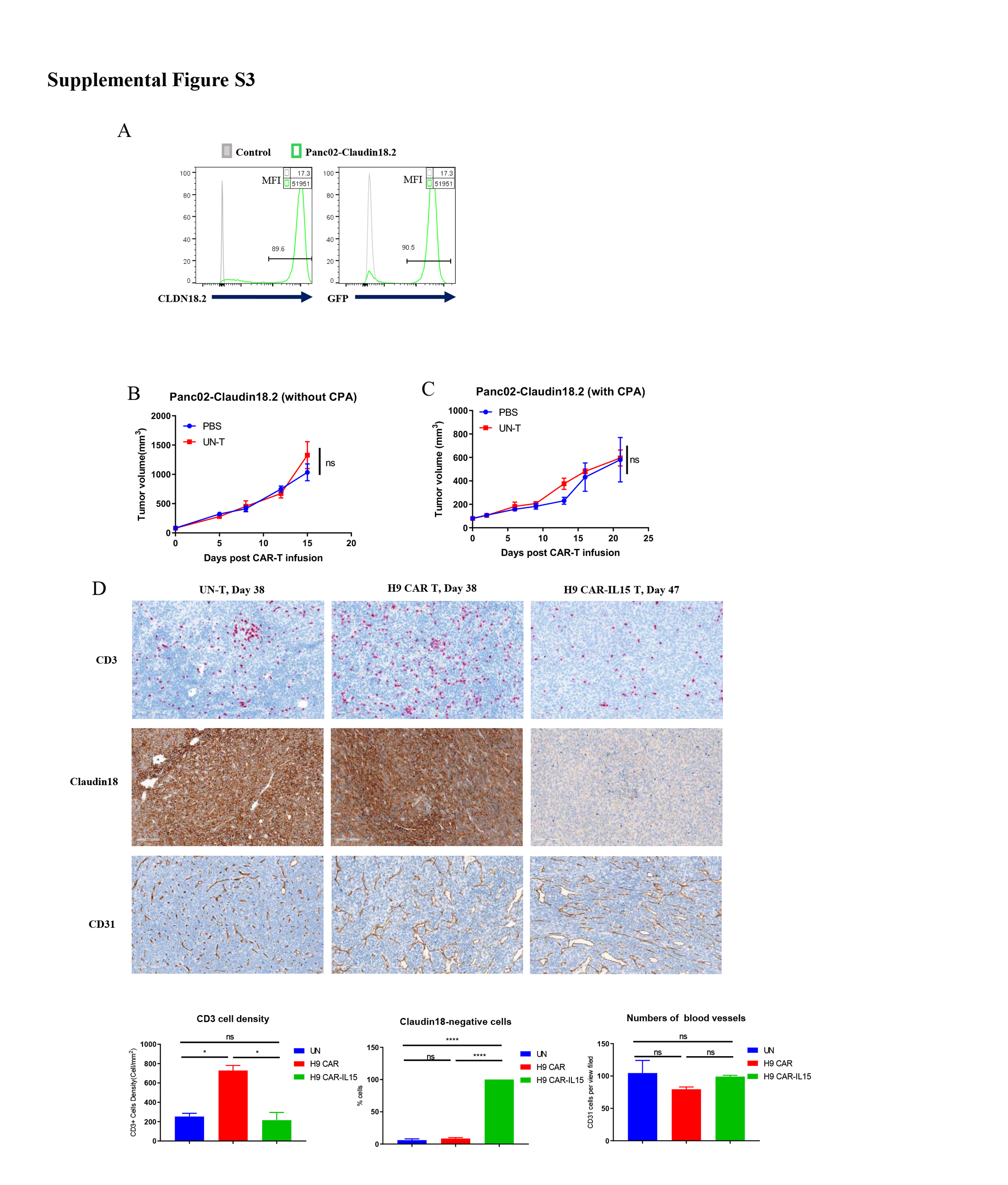

Supplement: Supplementary Figure 3 — (A) Representative flow cytometry plots showing the expression of Claudin 18.2 and GFP in ex vivo cultured Panc02-Claudin 18.2 cells after one in vivo tumour passage. (B, C) C57BL/6 mice were inoculated s.c Panc02-Claudin18.2 cells and treated with 5 x 10^6^ UN-T cells or PBS (same volume) in the absence (B) or presence (C) of CPA pretreatment. Tumour growth was assessed over time, and the results are expressed as the mean tumour volume (mm3 ± SD) with n = 5 mice per group. (D) Representative IHC images and statistical results (bottom panel) for the expression of CD3 (upper panel), CD31 (lower panel) and Claudin 18 (middle panel) in tumours from UN-T (left), H9 CAR (middle) and H9 CAR-IL15 (right)-treated mice on Days 38 or 47 after treatment. Statistical analyses were performed using one-way ANOVA with Tukey’s post hoc correction test (D) and two-way ANOVA (B, C). Statistical significance was defined as follows: ns, not significant, P > 0.05; *, P < 0.05; ****, P < 0.0001. [file Image_3.tif]

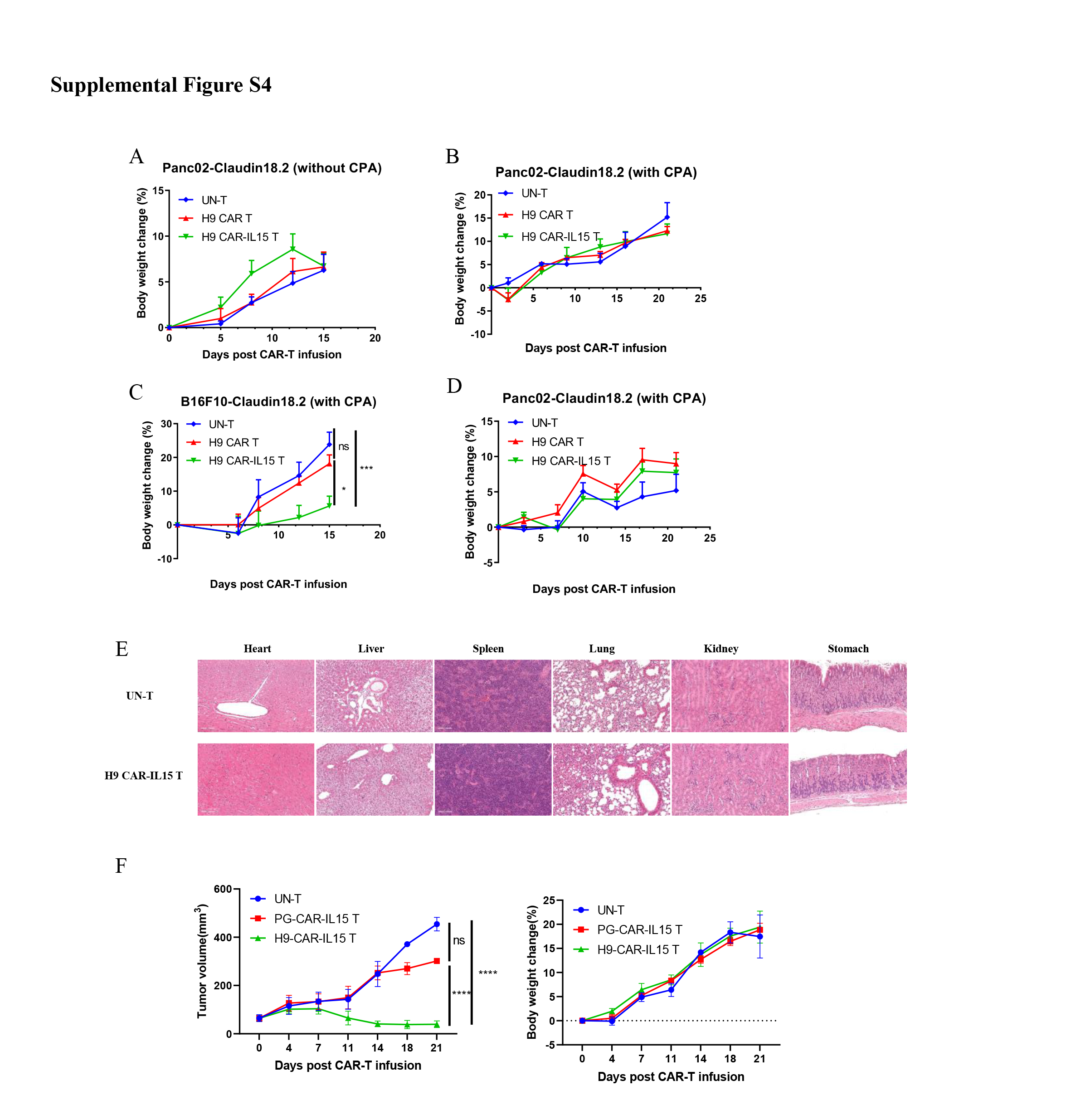

Supplement: Supplementary Figure 4 — (A-D) Body weight changes in treated mice over time were assessed. (E) Histopathological analysis of vital organ tissues by haematoxylin and eosin (H&E) staining of Panc02-Claudin18.2 xenografts pretreated with CPA. Mice were treated with CAR-T cells and sacrificed on Day 23 following T-cell infusion, and their vital organs were harvested, formalin-fixed, paraffin-embedded, and stained with H&E. Representative photomicrographs are shown. The images were taken at 200× magnification. (F) C57BL/6 mice were inoculated s.c. Panc02-Claudin18.2 cells and treated with CAR-T cells (2.5*106 cells) in the presence of CPA pretreatment. Tumour growth was assessed over time, and the results are expressed as the mean tumour volume (mm3 ± SD) with n = 5 mice per group (UN-T group, PG CAR-IL15 T group, H9 CAR-IL15 T group). Body weight changes in treated mice over time were also assessed. Statistical analyses were performed using one-way ANOVA with Tukey’s post hoc correction test (F) and two-way ANOVA (C). Statistical significance was defined as follows: ns, not significant, P > 0.05; *, P < 0.05; ***, P < 0.001. [file Image_4.tif]

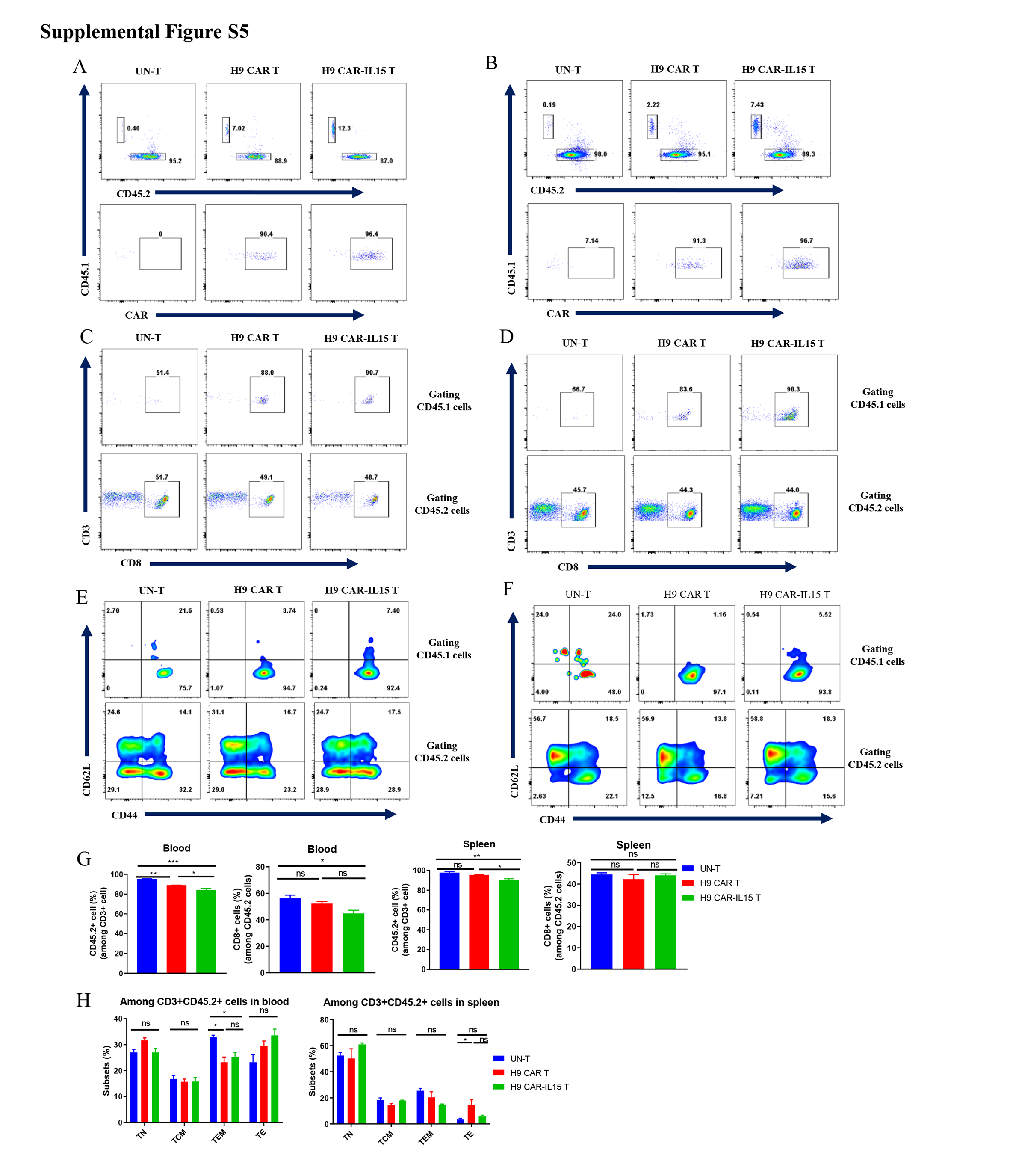

Supplement: Supplementary Figure 5 — (A, B) Representative flow cytometric plots showing the percentage of CD45.1+ adoptively transferred cells and CD45.2+ host endogenous and CAR-T cells in the blood (A) and spleen (B) from treated mice at Day 8 after CAR-T-cell treatment. (C, D) Representative flow cytometric plots showing the percentage of CD8+ CAR-T (gating CD45.1+ cells) or host endogenous (gating CD45.2+ cells) T cells in the blood (C) and spleen (D) from treated mice at Day 8 after CAR-T treatment. (E, F) Representative flow cytometric plots showing the expression of CD44 and CD62L on CAR-T (gating CD45.1+ cells) or host endogenous (gating CD45.2+ cells) T cells in the blood (E) and spleen (F) from treated mice at Day 8 after CAR-T treatment. (G) Bar graphs showing the percentage of host endogenous CD45.2+ T cells and CD8+ T cells in the blood (left) and spleen (right) from treated mice at Day 8 after CAR-T-cell treatment. (H) Bar graphs showing the percentage of host endogenous TN (CD44-CD62L+), TCM (CD44+CD62L+), TEM (CD44+CD62L-) and TE (CD44-CD62L-) subsets in the blood (left) and spleen (right) from treated mice at Day 8 after CAR-T-cell treatment. The data represent the mean ± SD of 3 mice per group. Statistical analyses were performed using one-way ANOVA with Tukey’s post hoc correction test (G, H). The significance of findings was defined as follows: ns, not significant; ns, P>0.05; *, P<0.05; **, P<0.01; ***, P<0.001. [file Image_5.tif]

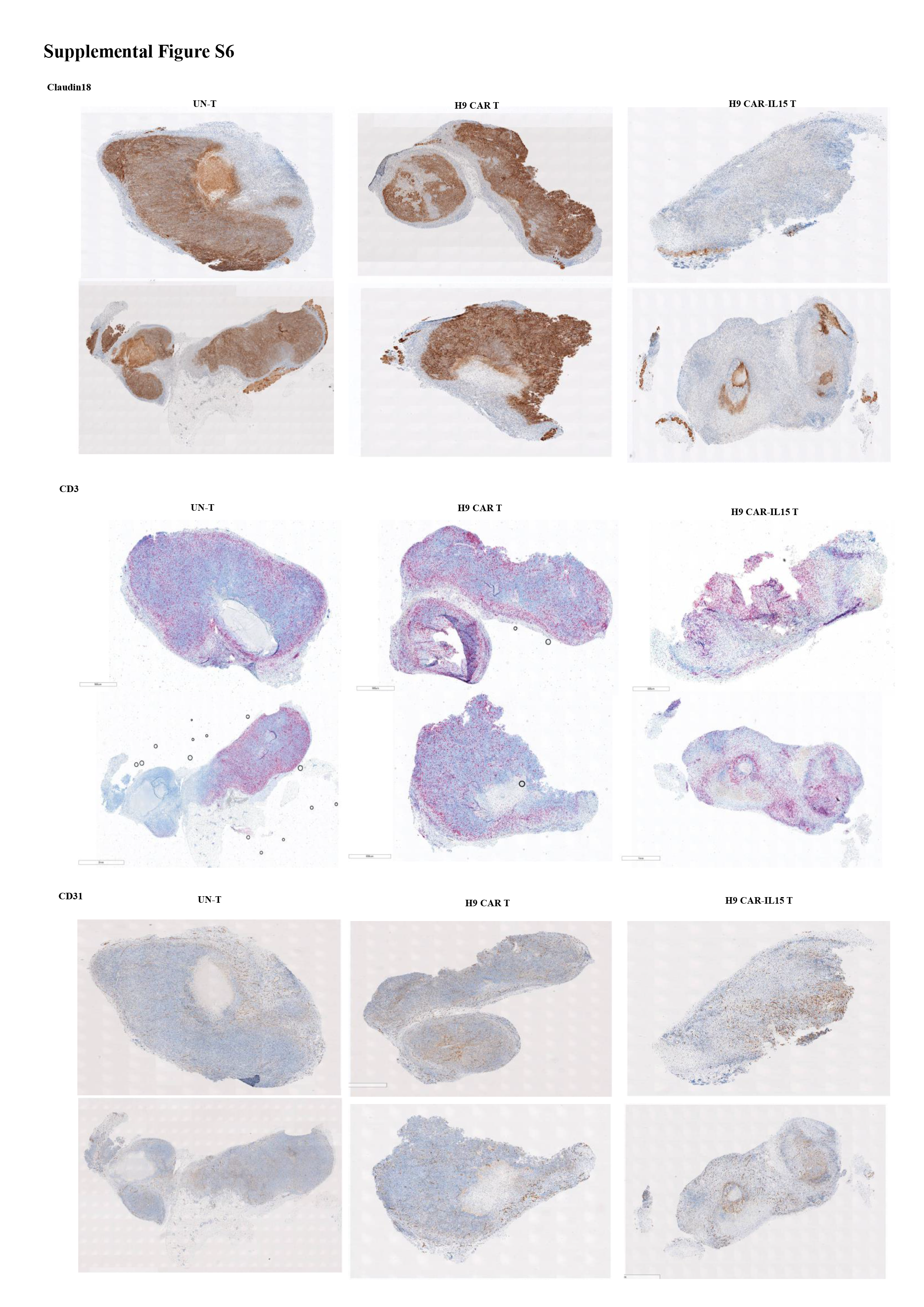

Supplement: Supplementary Figure 6 — Full IHC images of tumours with two tumours each group for analysis of CD3, Claudin18.2 and CD31 expression. [file Image_6.tif]

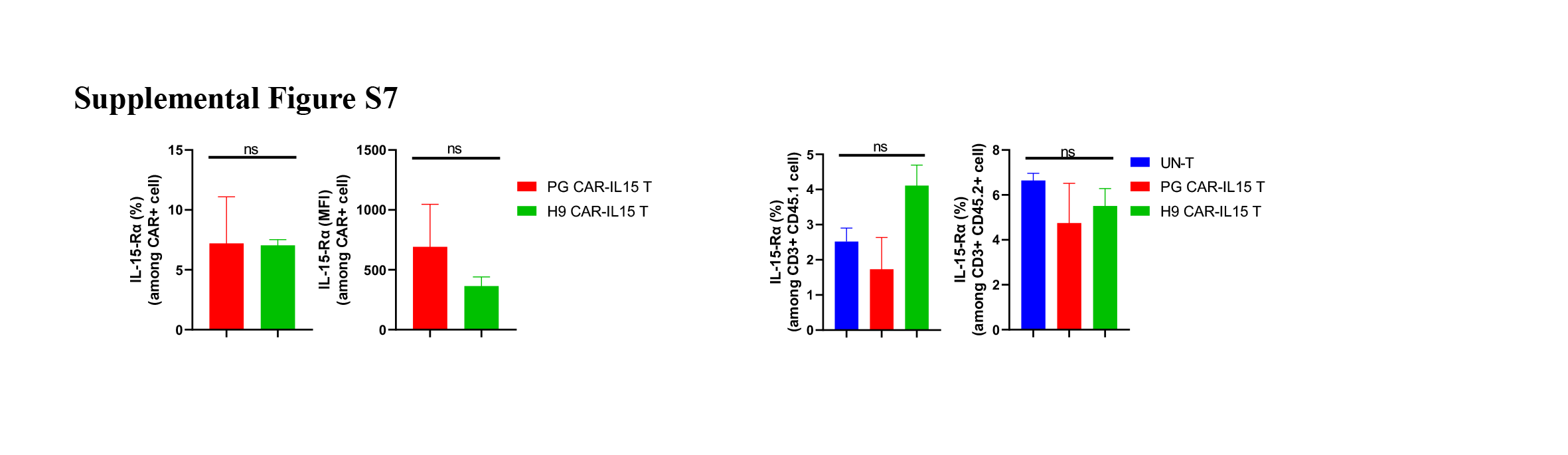

Supplement: Supplementary Figure 7 — The bar graphs showing the percentage and/or MFI of IL15Rα expressed on circulating CAR-T cells (CD3+CD45.1+CAR+ cells), total infused T cells (CD3+CD45.1+ cells) and host endogenous T cells (CD3+CD45.2+ cells) from treated mice at day 7 after treatment. The data represent mean ± SD of 3 mice per group. Statistical analyses were performed using a one-way ANOVA with Tukey post hoc correction test. [file Image_7.tif]

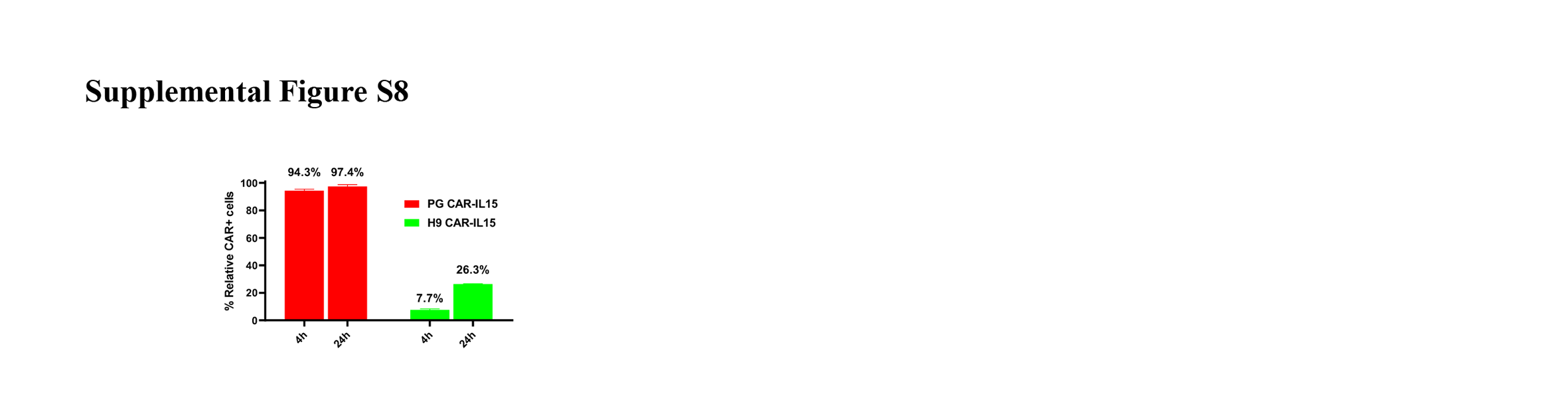

Supplement: Supplementary Figure 8 — Effector cells (H9 CAR-IL15 T or PG CAR-IL15 T) and target cells (Panc02-Claudin18.2 or Panc02-Claudin18.1) were cocultured at 1:1 E/T ratio, 4 hours and 24 hours later, the expression of CAR on effector cells was detected by flow cytometry. The bar graphs showing the mean of relative CAR expression after CAR engagement (cocultured with Panc02-Claudin18.2) at 4 hours and 24 hours normalized to that after no CAR engagement (cocultured with Panc02-Claudin18.1). Results show mean ± SD of triplicate wells. [file Image_8.tif]
